# Relationship between domains of physical activity and cardiac autonomic modulation in adults: a cross-sectional study

**DOI:** 10.1038/s41598-020-72663-7

**Published:** 2020-09-23

**Authors:** William R. Tebar, Raphael M. Ritti-Dias, Jorge Mota, Breno Q. Farah, Bruna T. C. Saraiva, Tatiana M. M. Damato, Leandro D. Delfino, Beatriz A. S. Aguilar, Amanda B. dos Santos, Stefany C. B. Silva, Luiz Carlos M. Vanderlei, Diego G. D. Christofaro

**Affiliations:** 1 School of Technology and Sciences, São Paulo State University (Unesp), Presidente Prudente, Brazil; 2 Universidade Nove de Julho (UNINOVE), Sao Paulo, Brazil; 3 Research Center on Physical Activity, Health and Leisure (CIAFEL), Faculty of Sport, University of Porto, Porto, Portugal; 4 Universidade Rural de Pernambuco, Recife, PE Brazil; 5 Department of Physical Education, São Paulo State University, Roberto Simonsen Street, Number 305, Presidente Prudente, 19060-900 Brazil

**Keywords:** Cardiology, Public health

## Abstract

This study aimed to analyze the relationship of physical activity in different domains with cardiac autonomic modulation in adults. A sample of 252 adults was randomly selected, with mean age of 42.1 (± 16.5) years, being 58% of women. Cardiac autonomic modulation was assessed through indexes of heart rate variability in time (SDNN, RMSSD) and frequency (LF, HF) domains for linear method, and by Poincaré plot for non-linear method (SD1, SD2 components). Domains of PA (occupation, sport, leisure time/commuting, and total) were assessed by Baecke’s questionnaire. Variables of age, gender, socioeconomic status (questionnaire) and body mass index (objectively measures) were covariates. Occupational PA was positively related to LF (β = 2.39, 95% CI 0.24; 4.54), sports PA was positively related to SDNN (β = 3.26, 95% CI 0.18; 7.05), RMSSD (β = 4.07, 95% CI 0.31; 7.85), and SD1 (β = 2.85, 95% CI 0.11; 5.81), and leisure time/commuting PA was positively related to SDNN (β = 3.36, 95% CI 0.28; 6.70) and RMSSD (β = 3.53, 95% CI 0.46; 7.52) indexes. Total PA was related to RMSSD (β = 1.70, 95% CI 0.04; 3.72). Sports, leisure time/commuting, and total PA were related to higher parasympathetic modulation, while occupational PA was related to higher sympathetic modulation to the heart in adults.

## Introduction

The cardiac autonomic modulation reflects the sympathetic and vagal activity for the heart. In rest, an increase in sympathetic modulation coupled with a reduction in vagal modulation has been associated with fatal and non-fatal cardiovascular events in adults^1–3^.

Previous studies have shown that cardiac autonomic modulation is enhanced with high levels of physical activity (PA), especially moderate-to-vigorous PA^4–7^. On the other hand, is unclear whether this association occurs in different domains of PA (occupation, sport, leisure time/commuting).

Different domains of physical activity have been differently associated to cardiovascular health and mortality^8–10^, besides other health indicators, as quality of life, where leisure time PA has been associated to better quality of life and occupational PA does not associate^11^. It was reported opposing effects of leisure time and occupational PA in global health of adult population, resulting in a health paradox^12^.

Moreover, it is important to investigate the specificity of each domain, mainly because the types of PA at different domains tend to be different with regard duration, intensity, frequency, and environment. This study aimed to investigate the hypothesis that leisure time, sport, and occupational domains of physical activity are differently related with cardiac autonomic modulation indicators in adults, regardless of gender, age, socioeconomic condition, and body mass index.

## Methods

This cross-sectional study follows the Strengthening the Reporting of Observational Studies in Epidemiology (STROBE) and was approved by the Ethical Research Committee of São Paulo State University under protocol CAAE: 72191717.9.0000.5402. All methods were performed in accordance with the relevant guidelines and regulations, as well as all the participants signed the Informed Consent Term agreeing to take part of the research, being informed about all procedures, without any costs, and free to desert of the research at any moment. The protocol of this research was previously registered at ClinicalTrials.gov, number NCT03986879.

The data collection occurred between December 2018 and June 2019, and the study was carried out in the city of Santo Anastacio, located in the southeastern region of Brazil, which has a population of ~ 16,000 adults and a Human Development Index of 0.753 (a measurement of life expectancy, education and per capita income, where 1 is the best and 0 the worse). Adults aged 18 years or over were randomly selected to participate in this study. To calculate the sample size, it was considered a correlation value of r = 0.23 for the relationship between PA and cardiac autonomic modulation based on previous studies^6^, a sampling power of 80%, and an alpha error of 5%, which totalized a minimum sample size of 147 subjects. However, predicting possible errors in the answer to the questionnaires or errors in the series of RR intervals from the cardiac autonomic modulation analysis, an additional 30% was added, resulting in 192 subjects. Besides that, the present study considered socioeconomic status, age, gender, and body mass index as adjustment variables, which resulted in the inclusion of 15 participants for each variable, resulting in a final sample size of 252 subjects.

Regarding the sample collection process, all the census sectors of the urban area of the city were considered (23 sectors), as well the number of inhabitants by sector, so the proportionality of each sector was considered (the proportion of population living in each sector was considered as the proportion of the sample to be assessed). Neighborhoods, streets and finally households of each census sector were randomized using the “Random” function of the SPSS statistical package. The detailed sample process was previously described in literature^13^.

For data collection, previously trained researchers visited the selected households and applied the questionnaire in those who agreed to participate in the research. After this procedure, the assessment of cardiac autonomic modulation performed by heart rate variability (HRV), was scheduled within the next seven days in a previously stipulated place.

### Physical activity

The habitual practice of PA was assessed by the Baecke’s questionnaire^14^, previously validated to Brazilian adults^15^, and with reliability tested against gold standard methods as doubly labeled water^16^. This instrument assesses PA through 16 questions in the last twelve months considering: physical effort at work (8 questions), the practice of sports activities or systematized exercises (4 questions), and physical activities at leisure time and commuting (4 questions). In domain of occupational activities, questions related to physical efforts at work are investigated, such as: time spent sitting, standing and walking in the work/occupational environment, carrying weights, frequency of perspiring a lot, and how tired feels after a workday. In sports domain, the practice of leisure-time exercises is considered, such as training at the gym or playing sports. In these domains, the intensity of these activities (light, moderate, vigorous), the number of hours per week in which these activities are practiced, and how long this physical activity has been practiced (< 1 month, 1–3 months, 4–6 months, 7–9 months, > 9 months). In the domain of leisure time and commuting, different activities in free time are considered, as well as the amount of time at commuting by walking and cycling to perform daily activities (i.e. for going to the market, shopping or going to work).

At the end, this instrument offers a dimensionless score for each domain, which ranges from 1 to 5, and the sum of the three scores corresponds to the total physical activity score.

### Cardiac autonomic modulation

The assessment of cardiac autonomic modulation was performed through HRV analysis. For this assessment, the subjects were instructed not to ingest alcoholic beverages and caffeinated drinks as coffee and tea, as well as not to perform moderate-to-vigorous intensity physical activity for a minimum period of 12 h so they could not cause changes in the autonomic modulation^17^. HRV was assessed by the Polar monitor, model V800 (Polar Electro Oy, Kempele, Finland), recorded beat-to-beat in a room with controlled temperature between 21 to 24° C and relative humidity between 50 to 60%^17^.

For HRV analysis, beat-to-beat heart rate was recorded for a period of thirty minutes, with participants lying in the supine position. In the series of RR intervals obtained, a digital filtering was performed and further complemented by manual filtering, aiming to eliminate premature ectopic beats. In this filtering, only series with more than 95% of sinus beats were considered and 1000 RR intervals of the most stable period of the tracing was used for analysis^18^. If any participant had less than 95% of sinus beats, the HRV recording and analysis were performed again.

The HRV analysis was performed using linear methods (time and frequency domains) and non-linear methods. For linear methods, frequency domain was analyzed by the Low Frequency (LF—0.04 to 0.15 Hz) and High Frequency (HF—0.15 to 0.4 Hz) spectral components, expressed in normalized units, and time domain was analyzed by using RMSSD and SDNN indexes. The RMSSD corresponds to the square root of the square mean of the differences between adjacent normal RR intervals in a time interval, while SDNN is the standard deviation of mean of all normal RR intervals, both expressed in milliseconds. The spectral analysis was calculated using the Fast Fourier transform algorithm^19^. For HRV analysis using non-linear methods, the Poincaré plot was quantitatively analyzed using the following indexes: SD1 (standard deviation of instantaneous beat-to-beat variability) and SD2 (long-term standard deviation of continuous R–R intervals).

For HRV analysis in linear and non-linear methods, Kubios HRV Analysis software version 2.0 (Kupio University, Finland—URL: www.kubios.com) was used.

### Socioeconomic condition

To assess the socio-economic condition, Brazilian Criteria for Economic Classification was used^20^. This instrument considers the level of education and the quantity of consumer goods and certain rooms in the household (i.e. bathrooms, cars, motorcycles, freezers, computers, washing machines, housekeeper), providing an specific scoring. The socioeconomic status was coded according to cutoff points of instrument, from higher to lower socioeconomic class, as follows: 1 = A class (45–100 points), 2 = B1 class (38–44 points), 3 = B2 class (29–37 points), 4 = C1 class (23–28 points), 5 = C2 class (17–22 points), and 6 = D–E classes (0–16 points).

### Anthropometry

Objective measurements of body mass and height were collected with participants’ barefoot and wearing light clothes. Body mass was obtained by a digital scale (OMRON HEALTHCARE Co., Ltd, Muko, Japan) with a maximum capacity of 180 kg and precision in 0.1 kg. The height was collected through a wall-mounted stadiometer (Wiso, Brazil) with a capacity of 2.2 m and precision in 0.1 cm. The values of body mass and height were used to calculate body mass index (BMI = body mass (kg)/height (m)^2^). For sample characterization, participants with BMI between 25 and 29.9 kg/m^2^ were classified as “overweight” and those participants with BMI equal or above 30 kg/m^2^ was classified as “obesity”, according to global classification for adult population^21^.

### Statistical analysis

Sample characterization variables were presented as mean and standard deviation, with gender differences compared by independent t test. The correlation between the different domains of physical activity and the indices of cardiac autonomic modulation were verified by Pearson's correlation. The magnitude of the relationships between the PA domains and cardiac autonomic modulation were observed by linear regression models, adjusted by age, gender, socioeconomic status, and body mass index. An dummy variable gender × domain-specific physical activity was used as adjustment for interaction between independent variables. The confidence interval adopted in the present study was 95% and the statistical significance was fixed in p < 0.05. The statistical package used was IBM SPSS Statistics for Windows, version 24.0 (IBM Corp., Armonk, N.Y., USA—URL: www.ibm.com/products/spss-statistics).

### Patient and public involvement

The patients and the public were not involved in the design, or conduct, or reporting, or dissemination plans of this research.

## Results

The sample of the present study (Table 1) comprised 252 participants, being 147 women and 105 men. The characteristics of sample are presented in Table 1.

**Table 1:** Sample characteristics.

Variable	Mean	Standard deviation
Age (years)	42.13	16.45
Body mass index (kg/m^2^)	28.13	5.26
**HRV indexes**		
SDNN (milliseconds)	48.25	20.97
RMSSD (milliseconds)	32.33	21.25
LF (normalized units)	63.33	16.32
HF (normalized units)	36.62	16.37
SD1	23.69	17.43
SD2	63.02	26.59
**Baecke’s physical activity scores**		
Occupational domain	2.87	1.05
Sport domain	2.46	0.83
Leisure time/commuting domain	2.33	0.73
Total score	7.67	1.76

*HRV* Heart rate variability, *SDNN* Standard deviation of the mean of all normal RR intervals, *RMSSD* Square root of the square mean of the differences between adjacent normal RR intervals in a time interval, *LF* Low frequency, *HF* High frequency, *SD1* Standard deviation of instantaneous beat-to-beat variability, *SD2* Long-term standard deviation of continuous R–R intervals, *PA* Physical activity.

The socioeconomic status was significantly correlated with physical activity, as better socioeconomic status were correlated to lower Occupational score (r = 0.129, p = 0.042) and higher Sport score (r = − 0.293, p = 0.001), whereas no significant correlation was observed between socioeconomic status and HRV indexes. The age was correlated with all HRV indexes at p < 0.01 level (SDNN r = − 0.361; RMSSD r = − 0.416; LF nu r = 0.210; HF nu r = − 0.197; SD1 r = − 0.383; SD2 = − 0.336). Body mass index was correlated with SDNN (r = − 0.208, p = 0.001), RMSSD (r = − 0.156, p = 0.015), and SD2 (r = − 0.220, p = 0.001). Mean difference between gender (men vs. women) were observed for HRV indexes of SDNN (52.2 ± 22.6 vs. 45.3 ± 19.2, p = 0.012), LF nu (66.4 ± 15.5 vs. 60.1 ± 16.5, p = 0.003), HF nu (34.0 ± 16.5 vs. 39.9 ± 16.1 p = 0.005), and SD2 (68.4 ± 29.1 vs. 58.9 ± 23.8, p = 0.007).

Table 2 shows the correlations between cardiac autonomic modulation and the different PA domains. Sport, leisure time/commuting, and total PA were positively related to SDNN, RMSSD, SD1 and SD2 indexes. Occupational PA was not correlated with cardiac autonomic modulation.

**Table 2:** Correlation between cardiac autonomic modulation and different domains of physical activity.

HRV indexes	Occupational PA		Sport PA		Leisure time/commuting PA		Total PA	
	r	p-value	r	p-value	r	p-value	r	p-value
SDNN (ms)	− 0.03	0.619	0.19	0.002	0.16	0.010	0.14	0.026
RMSSD (ms)	− 0.01	0.975	0.21	0.001	0.18	0.005	0.18	0.005
LF (nu)	0.09	0.148	− 0.11	0.094	− 0.08	0.186	− 0.03	0.627
HF (nu)	− 0.06	0.346	0.10	0.137	0.07	0.272	0.04	0.545
SD1	− 0.01	0.927	0.20	0.002	0.16	0.011	0.16	0.011
SD2	− 0.04	0.572	0.17	0.007	0.14	0.025	0.12	0.058

*HRV* Heart rate variability, *SDNN* Standard deviation of the mean of all normal RR intervals, *RMSSD* Square root of the square mean of the differences between adjacent normal RR intervals in a time interval, *LF* Low frequency, *HF* High frequency, *SD1* Standard deviation of instantaneous beat-to-beat variability, *SD2* Long-term standard deviation of continuous R–R intervals, *PA* Physical activity, *ms* milliseconds, *nu* normalized units.

Data of the multivariate analysis are presented in Tables 3 and 4. Sport PA was positively related with SDNN, RMSSD and SD1. Each increment in Sport PA score was associated with an increase of about 4 units of milliseconds in the RMSSD and 3 units of milliseconds in the SDNN, which corresponds to a positive relationship in parasympathetic modulation and global variability, respectively. Occupational PA was positively related to LF nu. Leisure time/commuting PA was positively related to SDNN and RMSSD, while Total PA was only positively related to RMSSD index in adjusted analysis.

**Table 3:** Relationship of cardiac autonomic modulation with physical activity at domains of occupation and sport among Brazilian adults (n = 252).

	β	Occupational PA							β	Sport PA						
		Unadjusted			β	Adjusted*				Unadjusted			β	Adjusted*		
		95% CI	Adj.R^2^	p-value		95% CI	Adj.R^2^	p-value		95% CI	Adj.R^2^	p-value		95% CI	Adj.R^2^	p-value
SDNN (ms)	− 0.62	− 3.11; 1.85	− 0.003	0.619	− 0.64	− 2.68; 2.55	0.116	0.962	**4.75**^†^	**1.70; 7.81**	**0.032**	**0.002**	**3.26**^†^	**0.18; 7.05**	**0.135**	**0.039**
RMSSD (ms)	0.44	− 2.70; 2.79	− 0.004	0.975	0.48	− 2.39; 3.35	0.091	0.741	**5.82**	**2.45–9.18**	**0.040**	**0.001**	**4.07**	**0.31; 7.85**	**0.111**	**0.034**
LF (nu)	1.42	− 0.50; 3.34	0.004	0.148	**2.39**^†^	**0.24; 4.54**	**0.079**	**0.030**	− 2.05	− 4.47; 0.35	0.007	0.094	1.26	− 4.16; 1.64	0.064	0.393
HF (nu)	− 0.94	− 2.89; 1.01	0.000	0.346	− 1.62	− 3.84; 0.60	0.062	0.151	1.85	− 0.59; 4.29	0.005	0.137	1.08	− 1.87; 4.04	0.054	0.470
SD1	0.0.9	− 2.17; 1.98	− 0.004	0.927	0.18	− 2.06; 2.43	0.075	0.871	**4.12**	**1.57; 6.68**	**0.035**	**0.002**	**2.85**	**0.11; 5.81**	**0.091**	**0.049**
SD2	− 0.91	− 4.08; 2.26	− 0.003	0.572	− 0.15	− 2.58; 3.27	0.104	0.929	**5.40**^†^	**1.49; 9.32**	**0.025**	**0.007**	4.27	− 0.24; 8.78	0.119	0.063

Bold indicates domains that are statistically related to cardiac autonomic modulation.

*PA* Physical activity, *CI* Confidence interval, *Adj.R*^2^ Adjusted R squared, *SDNN* Standard deviation of the mean of all normal RR intervals, *RMSSD* Square root of the square mean of the differences between adjacent normal RR intervals in a time interval, *LF* Low frequency, *HF* High frequency, *SD1* Standard deviation of instantaneous beat-to-beat variability, *SD2* Long-term standard deviation of continuous R-R intervals, *ms* milliseconds, *nu* normalized units.

*Adjusted by gender, age, socioeconomic level, body mass index.

^†^Statistical model with significant interaction between gender × physical activity.

**Table 4:** Relationship of cardiac autonomic modulation with physical activity at leisure time/commuting domain and in total among Brazilian adults (n = 252).

	β	Leisure time/commuting PA							β	Total PA						
		Unadjusted			β	Adjusted*				Unadjusted			β	Adjusted*		
		95%CI	Adj.R^2^	p-value		95%CI	Adj.R^2^	p-value		95%CI	Adj.R^2^	p-value		95%CI	Adj.R^2^	p-value
SDNN (ms)	**4.61**^†^	**1.12–8.11**	**0.022**	**0.010**	**3.36**	**0.28; 6.70**	**0.132**	**0.048**	**1.67**^†^	**0.20; 3.13**	**0.016**	**0.026**	1.36	− 0.21; 2.94	0.130	0.090
RMSSD (ms)	**5.56**	**1.71–9.42**	**0.027**	**0.005**	**3.53**	**0.46; 7.52**	**0.104**	**0.043**	**2.31**	**0.70; 3.93**	**0.027**	**0.005**	**1.70**	**0.04; 3.72**	**0.107**	**0.045**
LF (nu)	− 1.85	− 4.60; 0.90	0.003	0.186	− 1.40	− 4.46; 1.66	0.064	0.367	− 0.29	− 1.44; 0.87	− 0.003	0.627	0.28	− 1.04; 1.61	0.061	0.672
HF (nu)	1.56	− 1.22; 4.34	0.001	0.272	1.10	− 2.02; 4.22	0.054	0.489	0.36	− 0.81; 1.52	− 0.003	0.545	− 0.16	− 1.51; 1.19	0.052	0.811
SD1	**3.81**	**0.79; 3.83**	**0.022**	**0.011**	2.34	− 0.78; 5.47	0.085	0.141	**1.60**†	**0.37; 2.84**	**0.022**	**0.011**	1.13	− 0.24; 2.50	0.087	0.106
SD2	**5.12**†	**0.65; 9.60**	**0.016**	**0.025**	3.88	− 0.88; 8.64	0.115	0.109	1.84	− 0.06; 3.73	0.010	0.058	1.60	− 0.49; 3.69	0.114	0.132

Bold indicates domains that are statistically related to cardiac autonomic modulation.

*PA* Physical activity, *CI* Confidence interval, *Adj.R*^2^ Adjusted R squared, *SDNN* Standard deviation of the mean of all normal RR intervals, *RMSSD* Square root of the square mean of the differences between adjacent normal RR intervals in a time interval, *LF* Low frequency, *HF* High frequency, *SD1* Standard deviation of instantaneous beat-to-beat variability, *SD2* Long-term standard deviation of continuous R-R intervals, *ms* milliseconds, *nu* normalized units.

*Adjusted by gender, age, socioeconomic level, body mass index.

^†^Statistical model with significant interaction between gender × physical activity.

The interaction term gender × physical activity was significant related in the models of Occupational PA vs. LF, Sport PA vs. SDNN and SD2, Leisure time/Commuting PA vs. SDNN and SD2, and of Total PA vs. SDNN and SD1 presented in Tables 3 and 4. According to gender, Occupational PA vs. LF was significant only in females (β = 2.87, p = 0.029); Sports PA was related with SDNN only in males (β = 5.27, p = 0.032) and its relationship with SD2 lost statistical significance for both males and females; Leisure time/Commuting PA was significant related with SDNN (β = 6.29, p = 0.023) and with SD2 (β = 7.24, p = 0.047) only in males; and Total PA was related with SDNN (β = 3.43, p = 0.008) and SD1 (β = 3.26, p = 0.006) only in males.

## Discussion

This study showed different associations between domains of physical activity with cardiac autonomic modulation in adult population. Sport and Leisure time/commuting were related to higher parasympathetic modulation to the heart, being evident in several HRV indexes.

It was observed that occupational PA score was positively related with LF index of HRV in the present study. Occupational activities could be, in general, less intense than physical activities in other domains, as well as may be susceptible to exposure of environmental conditions related to work, as physical and mental stress, worries, and work overload. Besides that, high levels of job stress were associated with increased heart rate and decreased HRV indexes^22^. In this sense, this relationship could be a subject to further additional investigation.

Another important aspect to highlight is that LF index corresponds to a contradictory interpretation in cardiac autonomic modulation^23^. Traditionally, LF index was considered as a reflection of the sympathetic nervous system activity^24^, but a more recent study considered LF as an index of baroreflex function in the modulation of cardiac autonomic outflows, not being a measure of cardiac sympathetic tone^25^. Furthermore, a study with animal model reported that LF was neither a robust index of cardiac sympathetic nerve activity nor of baroreflex sensitivity, but highlight a link between them^26^.

PA sport domain was positively related to RMSSD, SDNN, and SD1 indexes in adjusted analysis in this study, which corresponds to higher modulation of parasympathetic nervous system and global variability^19^. While RMSSD represents parasympathetic modulation by estimating HRV changes mediated by vagal tone^27^, variations in SDNN index does not have distinction whether changes are related to sympathetic or vagal tone^28^. In turn, despite being considered as an identical metric to the RMSSD^29^, SD1 index represents a short-term HRV measurement which is correlated to baroreflex sensitivity^30^ and predicts a set of different factors which may reflect the complexity of mechanisms in HRV regulation, as diastolic blood pressure and HRV in time and frequency domains^31^. This increase in parasympathetic activity could be related to the vigorous-intensity of the great part of sport activities (higher than 6 METs)^32^, once it has been observed an increase in RMSSD index according to the intensity of physical activity^4^. Schmidt et al.^33^ observed that sports activity showed higher relationship with physical fitness and physical health status than habitual activity in 18 years of follow up. Based upon these evidences, it is hypothesized that high-intensity training may result in a positive change in the vagal activity^34,35^.

Physical activities in leisure time and commuting were positively related to RMSSD and SDNN indexes in the present study, regardless of gender, age, socioeconomic status, and body mass index. Likewise, Bueno et al.^36^ observed that adults that cycling for transportation presented higher parameters of parasympathetic modulation and lower parameters of sympathetic modulation than insufficiently active adults. Soares-Miranda et al.^37^ reported that higher leisure time physical activity, walking distance, and walking pace were associated to higher HRV indexes in older adults. The findings of the present study also raise the hypothesis that the time spent in leisure time and commuting activities take place from the time spent in sedentary behavior. Delfino et al.^38^ observed that those adults with high breaks in sedentary behavior at leisure and occupation showed high levels of habitual physical activity. In this sense, higher levels of sedentary behavior may result in less light-intensity physical activity, which may negatively impact cardiac autonomic modulation, once sedentary lifestyle was associated to lower RMSSD index and higher sympathovagal balance in adults^39^.

The present study also showed a positive relationship between total PA and RMSSD after adjusting for variables of interest. Corroborating with this finding, Tornberg et al.^4^ observed a positive relationship between self-reported PA and RMSSD index regardless of adiposity levels, and Soares-Miranda et al.^7^ reported that the most active group showed higher levels of vagal HRV indexes than the less active group in adult population. It is important to highlight that total PA in this study was a sum of the three different domains assessed by Baecke’s questionnaire, which were composed by different constructs in its scoring, as weekly frequency, perceived intensity, duration and how long these activities were performed, being composed by domains which was more related to HRV indexes than others, which could mitigate this relationship when analyzed in total score. Therefore, the dimensionless of Baecke score did not allow comparisons between physically actives and insufficiently actives according to global recommendations, or even inferences about how much intensity of amount of habitual PA in the assessed domains were related to cardiac autonomic modulation.

Even the analysis adjusted by potential confounding factors, the interaction between gender and physical activity may limit the present study findings. Previous study reported a significant gender difference in the activity of autonomic nervous system^40^ and was observed that men presented a predominance of sympathetic vascular regulation, while women showed a dominant influence of parasympathetic heart rate regulation^41^. In this sense, further investigation with gender-specific analysis is suggested.

This study has some limitations. It is important to highlight the observational design of this study, which does not allow cause and effect inferences. Further, the assessment of physical activity by questionnaire was susceptible to bias of memory and classification of intensity. Besides that, the use of medicines for cardiovascular control, the hour of HRV assessment, and menstrual cycle of women were not controlled. However, the representative sample randomly selected and analysis adjusted by potential confounding factors (gender, age, socioeconomic status, and body mass index) were positive strengths of the study. Besides that, the use of a questionnaire for physical activity allows to analyze its practice in different domains, which was the main purpose of this study, and would not be provided by accelerometer.

In conclusion, the association between physical activity and cardiac autonomic modulation in adults is dependent on the domain analyzed. Sport, leisure time/commuting, and total PA were positively related to parasympathetic modulation indexes, whereas occupational PA was related with sympathetic modulation indexes. As practical application, the stimulation of practice of PA in different domains, mainly in regard sports practice and leisure time/commuting activities may contributes to improvement in cardiac autonomic modulation in adult population, regardless gender, age, socioeconomic status, and body mass index.
